# Lymphocytic Choriomeningitis Virus in Person Living with HIV, Connecticut, USA, 2021

**DOI:** 10.3201/eid2909.230087

**Published:** 2023-09

**Authors:** Jonathan Dyal, Shiv Gandhi, Caitlin M. Cossaboom, Austin Leach, Ketan Patel, Marjorie Golden, Joseph Canterino, Marie-Louise Landry, Debi Cannon, Mary Choi, Inna Krapiunaya, John D. Klena, Trevor Shoemaker

**Affiliations:** Centers for Disease Control and Prevention, Atlanta, Georgia, USA (J. Dyal, C.M. Cossaboom, A. Leach, K. Patel, D. Cannon, M. Choi, I. Krapiunaya, J.D. Klena, T. Shoemaker);; Yale School of Medicine, New Haven, Connecticut, USA (S. Gandhi, M. Golden, J. Canterino, M. Landry)

**Keywords:** Lymphocytic choriomeningitis virus, HIV, Connecticut, United States, arenavirus, rodent-borne, zoonoses, viruses

## Abstract

Lymphocytic choriomeningitis virus is an underreported cause of miscarriage and neurologic disease. Surveillance remains challenging because of nonspecific symptomatology, inconsistent case reporting, and difficulties with diagnostic testing. We describe a case of acute lymphocytic choriomeningitis virus disease in a person living with HIV in Connecticut, USA, identified by using quantitative reverse transcription PCR.

Lymphocytic choriomeningitis virus (LCMV) is a globally occurring, Old World arenavirus. The common house mouse (*Mus musculus*) is the primary reservoir, although the virus can infect many other rodent species, including wild or domesticated rats, gerbils, and hamsters (*1*). In humans, LCMV infection is frequently asymptomatic but can present as a nonspecific viral illness, sometimes accompanied by headache or photophobia (*2*). Congenital LCMV infection carries a high risk for spontaneous abortion or serious neurologic deficits in the developing fetus. Children born after congenital LCMV infection frequently suffer from chorioretinitis, hydrocephalus, and psychomotor delay (*3*). Immunocompromised persons are also at greater risk for complications following LCMV infection because they can develop life-threatening encephalitis, seizures, and paralysis. Other groups at heightened risk for infection include transplant recipients and persons that work with rodents, such as laboratory staff and pet store workers (*4*,*5*). Because of its a broad geographic distribution and potential for severe disease, clarifying the effects of LCMV on human health remains an important public health challenge.

Because of its nonspecific symptoms, lack of physician awareness, suboptimal diagnostic testing, and limited and inconsistent reporting requirements, LCMV is an underrecognized public health threat. In the United States, Wisconsin is the only state that requires hospitals and healthcare providers to report cases of LCMV (*6*). Because LCMV is not nationally notifiable, the Centers for Disease Control and Prevention (CDC) receives case reports on a voluntary basis (*7*). Although outbreaks of LCMV have been identified, sporadic cases are likely underreported, and the true burden within the United States is unknown.

Laboratory diagnosis of an acute case of LCMV is made by detection of viral nucleic acids by using real-time reverse transcription PCR (qRT-PCR) and detection of circulating LCMV IgM or a rising titer of LCMV IgG. However, few commercial laboratories offer testing services for LCMV. LCMVs causing human infection are genetically diverse, a feature that has made the design and implementation of nucleic acid–based assays challenging (*8*). Taking advantage of improved reagents for PCR and an increase in the number of complete genomes available for analysis, we developed a qRT-PCR assay for LCMV, targeting the large segment. We describe a case of LCMV infection in a patient with well-controlled HIV, diagnosed by using the qRT-PCR and IgM and IgG enzyme-linked immunosorbent assays (ELISA) at CDC. Use of the qRT-PCR can accelerate detection of acute LCMV infection and therefore has the potential to improve patient care.

## The Case

A 53-year-old man residing in Connecticut, USA, with a history of well-controlled HIV and receiving antiretroviral therapy but with a chronically low CD4 count (150/µL) and percentage (14%), was admitted to the emergency department at Yale New Haven Hospital (New Haven, CT, USA) with a 2-day history of headache, nausea, and emesis. In the weeks leading up to admission, he had noted mice in his home. Approximately 2 weeks before admission, he cleaned mouse feces and urine with a vacuum while wearing gloves and a mask. At arrival to the hospital, he was febrile to 100.7°F but otherwise hemodynamically stable. Physical examination showed no neurologic deficits or nuchal rigidity. Laboratory data were notable for a hemoglobin of 11.2 g/dL (reference range 13.1–17.5), leukocyte count of 7,200/µL (reference range 4,000–10,000/µL), platelet count of 213,000/µL (reference range 150,000–400,000/µL), aspartate transaminase 59 U/L (reference range 10–35 U/L), and alanine aminotransferase 96 U/L (reference range 9–59 U/L). His HIV viral load was below the limit of detection (20 copies/mL) 1 month before admission.

The patient underwent a lumbar puncture after receiving empiric antibiotics for meningitis. Analysis of the cerebral spinal fluid (CSF) revealed a nucleated cell count of 500/µL in tube 1 and 495/µL in tube 4 (reference range 0–5/µL), which was lymphocyte predominant (≈85%). CSF protein was 116 mg/dL (reference range 15–45 mg/dL) and glucose was 66 mg/dL (reference range 40–70 mg/dL). Culture remained sterile and CSF was negative for herpes simplex virus by real-time PCR, West Nile virus IgM in CSF, and for 14 pathogens included on the BioFire FilmArray Meningitis/Encephalitis Panel (BioFire Diagnostics, LLC, https://www.biofiredx.com).

Because of concern for aseptic meningitis caused by LCMV, we sent CSF and whole blood samples to CDC for LCMV testing. LCMV IgM and IgG were determined by a CDC-developed ELISA, as previously described (*2*). For nucleic acid testing, in brief, samples were inactivated by using MagMAX Pathogen RNA/DNA Kit (ThermoFisher, https://www.thermofisher.com) and extracted on the KingFisher Duo Prime platform (ThermoFisher). Extracted samples were tested by using an qRT-PCR targeting the large segment of LCMV. RNA from samples positive by the LCMV qRT-PCR were selected for sequencing and subsequent phylogenetic analysis. RNA library sequencing, amplicon-based sequencing, and phylogenetic analysis were performed on the LCMV genome obtained from the CSF sample, as were additional laboratory methods (Appendix).

**Figure 1:**
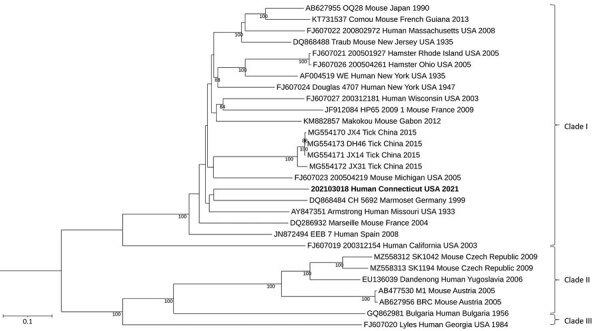
Maximum-likelihood analysis of the full large genome segment of lymphocytic choriomeningitis virus (LCMV) sample from a patient in Connecticut, USA (bold), compared with reference sequences. Branch nodes provide the bootstrap support values, as a percentage. Clades are indicated at right, and GenBank accession numbers are provided for reference sequences. Scale bar indicates number of substitutions per site.

**Figure 2:**
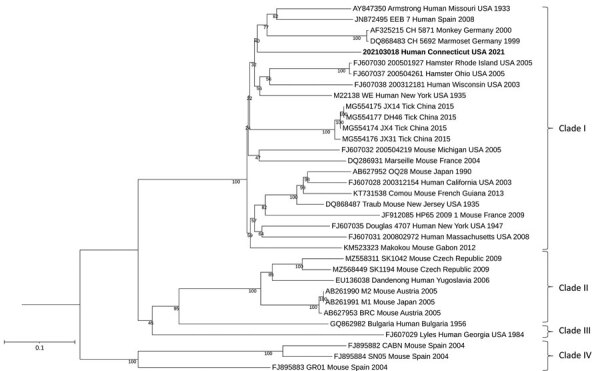
Maximum-likelihood analysis of the full small genome segment of lymphocytic choriomeningitis virus (LCMV) sample from a patient in Connecticut, USA (bold), compared with reference sequences. Branch nodes provide the bootstrap support values, as a percentage. Clades are indicated at right, and GenBank accession numbers are provided for reference sequences. Scale bar indicates number of substitutions per site.

Analysis of the CSF showed that LCMV IgM and IgG ELISA results were both negative, but qRT-PCR results were positive. On blood collected 3 days after the CSF sample, LCMV IgM and IgG ELISA results were both positive (titer ≥1:400), and qRT-PCR results were negative. Sequence analysis of both the small and large segments showed the LCMV strain clustered to lineage I (Figures 1, 2). After confirming a diagnosis of LCMV meningitis in the patient, we discontinued empiric antibiotics and discharged him on hospitalization day 10. The patient’s headaches resolved approximately 5 days after discharge, and he made a complete recovery.

## Conclusions

Despite minimal reporting requirements, LCMV is believed to be widespread throughout the United States and the world. As in the case we report, clinicians should maintain a high index of suspicion for LCMV infection in patients with identified rodent exposures and symptoms consistent with meningitis, especially in high-risk groups such as pregnant women and the immunocompromised. LCMV viremia occurs early and is transient but may seed the central nervous system. Thus, as occurred for the patient in this report, viral RNA may no longer be detectable in blood but may be detectable in CSF in patients with clinical signs and symptoms of LCMV. Consequently, for patients with suspected LCMV infection, parallel molecular and serologic testing of both blood and CSF is likely beneficial for early diagnosis and management.

By sequencing the full genome of an LCMV strain isolated from this patient and conducting a phylogenetic analysis, we have gained valuable insights into the evolutionary relationships and genetic diversity of LCMV. The obtained phylogenetic trees provide evidence for the relatedness of the patient's strain to other known LCMV strains and contributes to our understanding of the virus’s epidemiology. The LCMV strains we obtained group in clade I, which corresponds to the *M. musculus domesticus* host subspecies (*1*) and contains viruses from Asia, Europe, America, and Africa. The phylogenetic tree also shows that the strain identified in this patient was genetically distinct from strains that have been identified from other parts of the world.

No-cost molecular and serologic testing for LCMV is available to hospitals and health departments through CDC’s Viral Special Pathogens Branch (Division of High-Consequence Pathogens and Pathology, National Center for Emerging and Zoonotic Infectious Diseases). Increasing both community and healthcare provider awareness of LCMV and public health case reporting could improve surveillance efforts and clarify the true burden, risk factors, and distribution of LCMV infection in the United States.

Appendix Additional information for lymphocytic choriomeningitis virus in person living with HIV, Connecticut, USA, 2021.
