# Supplementary material for: Lymphocytic Choriomeningitis Virus in Person Living with HIV, Connecticut, USA, 2021
Source: Emerg Infect Dis. 2023 Sep;29(9):1886–9. doi: 10.3201/eid2909.230087 (PMC10461659; doi:10.3201/eid2909.230087)
Supplement: Appendix — Additional information for lymphocytic choriomeningitis virus in person living with HIV, Connecticut, USA, 2021. [file 23-0087-Techapp-s1.pdf]

Article DOI: http://doi.org/10.3201/eid2909.230087

EID cannot ensure accessibility for supplementary materials supplied by authors. Readers who have difficulty accessing supplementary content should contact the authors for assistance.

Lymphocytic Choriomeningitis Virus in Person Living with HIV, Connecticut, USA, 2021

Appendix

Methods

Ribonucleic acid extraction and RT-qPCR

Suspect LCMV sample was received as a clinical sample in whole blood and CSF matrix. Patient samples were extracted for total nucleic acids using the MagMAX Pathogen RNA/DNA kit (Applied Biosystems, 4462359) and the KingFisher Duo Prime Purification System (Thermo Scientific, 5400110). The extracted samples were tested for presence of LCMV RNA using an RT-qPCR assay designed to detect strains known to cause infections in humans. The LCMV RT-qPCR assay was developed by aligning complete LCMV genomes available in NCBI and targets the L segment between bases 3604 and 3770. It is multiplexed with an in-house human B2M housekeeping gene assay to assess sample integrity. The sample was tested in single replicate using the Luna® Probe One-Step RT-qPCR 4X Mix with UDG kit (NEB, M3019S). Cycle threshold values (C_t) of less than 40 are considered positive and further confirmed with sequencing data.

Illumina Library Construction

Samples positive by the LCMV RT-qPCR assays were selected for RNA-seq. Illumina libraries were prepared using the NEBNext® Ultra II RNA Library Prep Kit for Illumina® (NEB, E7770S). Ribosomal RNA depletion was performed using the RiboCop rRNA Depletion Kits for Human/Mouse/RatV1.3 (Lexogen, 037). RNA Shearing (fragmentation) was performed using

M220 Focused-ultrasonicator (Covaris, 500295). Fragmented rRNA depleted RNA was used as input for Illumina library construction.

Libraries were quantitated and normalized using Qubit dsDNA HS Assay Kit (Invitrogen, Q32851). The library was run on MiniSeq System (Illumina) using the MiniSeq High Output Reagent Kit (300-cycles). Geneious Prime 2020.2.2 was used to generate consensus LCMV genome using a map to reference strategy. To fill remaining gaps in the consensus LCMV genome, tilling primers against the S and L segments were designed using primalscheme with the consensus sequence obtained by Geneious Prime 2020.2.2 as the reference genome (*1*). Amplicon libraries generated using the tilling primers were sequenced on the MiSeq System (Illumina) using the MiSeq Reagent Kit v2 (500-cycles). Geneious Prime 2020.2.2 was used to generate consensus LCMV genome using a map to reference strategy.

Phylogenetic analysis

Phylogenetic analysis was performed using the consensus sequence obtained from the CSF whole genome sequencing and amplicon-based sequencing data. LCMV full length nucleotide sequences available from NCBI were aligned using MAFFT on Geneious Prime 2022.0.2 and analyzed by the IQTREE maximum-likelihood software to produce bootstrapped, midpoint-rooted trees. iTOL (version 6.5.8) was used to visualize and display the phylogenetic tree (*2,3*).

References

1. Kalyaanamoorthy S, Minh BQ, Wong TKF, von Haeseler A, Jermiin LS. ModelFinder: fast model selection for accurate phylogenetic estimates. Nat Methods. 2017;14:587–9. PubMed https://doi.org/10.1038/nmeth.4285

2. Nguyen L-T, Schmidt HA, von Haeseler A, Minh BQ. IQ-TREE: a fast and effective stochastic algorithm for estimating maximum-likelihood phylogenies. Mol Biol Evol. 2015;32:268–74. PubMed https://doi.org/10.1093/molbev/msu300

3. Hoang DT, Chernomor O, von Haeseler A, Minh BQ, Vinh LS. UFBoot2: Improving the ultrafast bootstrap approximation. Mol Biol Evol. 2018;35:518–22. PubMed https://doi.org/10.1093/molbev/msx281
